# Matrix Metalloproteinases (MMPs) and Inhibitors of MMPs in the Avian Reproductive System: An Overview

**DOI:** 10.3390/ijms22158056

**Published:** 2021-07-28

**Authors:** Anna Hrabia

**Affiliations:** Department of Animal Physiology and Endocrinology, University of Agriculture in Krakow, Al. Mickiewicza 24/28, 30-059 Krakow, Poland; anna.hrabia@urk.edu.pl or rzhrabia@cyf-kr.edu.pl

**Keywords:** MMPs, TIMPs, remodeling, follicle, ovary, oviduct, birds

## Abstract

Many matrix metalloproteinases (MMPs) are produced in the mammalian reproductive system and participate in the regulation of its functions. In birds, the limited information available thus far indicates that MMPs are significant regulators of avian ovarian and oviductal functions, too. Some MMPs and inhibitors of MMPs are present in the hen reproductive tissues and their abundances and/or activities change according to the physiological state. The intraovarian role of MMPs likely includes the remodeling of the extracellular matrix (ECM) during folliculogenesis, follicle atresia, and postovulatory regression. In the oviduct, MMPs are also involved in ECM turnover during oviduct development and regression. This study provides a review of the current knowledge on the presence, activity, and regulation of MMPs in the female reproductive system of birds.

## 1. Introduction

Growing evidence demonstrates that matrix metalloproteinase system elements, including metalloproteinases (MMPs) and inhibitors of MMPs, are produced in ovarian and oviductal tissues in birds. They can participate in the remodeling of the extracellular matrix (ECM) and in the modulation of cell functions such as proliferation, apoptosis, and differentiation. Thus, they can participate in cellular and extracellular events across folliculogenesis, follicle atresia, and postovulatory regression as well as during oviduct development and regression. This study provides a brief review of the current knowledge regarding the expression, activity, and regulation of MMPs in the hen reproductive system. The possible engagement of MMPs in local regulation of the hen reproductive functions is also discussed.

## 2. Characteristics of the Avian Ovary and Oviduct

**Figure 1:**
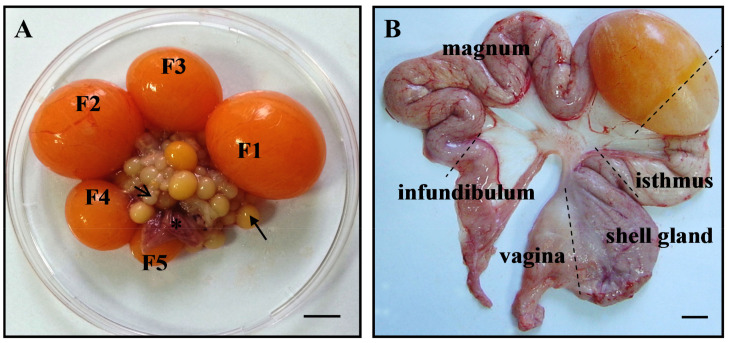
The ovary of an egg-laying hen (**A**). Explanations: white follicle (1–4 mm in diameter; open arrow), yellowish follicle (4–8 mm; arrow), yellow preovulatory follicles (F5–F1; 9–40 mm), postovulatory follicle (asterisk). The oviduct of a laying hen (**B**). Scale bars = 1 cm.

In the majority of female birds, only the left ovary and oviduct develop into functional organs. The laying domestic hen ovary is composed of follicles at different stages of development (Figure 1A). There are numerous slow-growing prehierarchical follicles (primordial, primary, white, and yellowish; up to 8 mm in diameter) and several rapidly growing yellow preovulatory follicles (9–40 mm) arranged in a distinct hierarchy, as well as several prehierarchical atretic follicles and postovulatory follicles (POFs) at different stages of regression. The largest yellow follicle, designated as F1, is the most mature and ovulates first. In laying hens, the follicle number in the preovulatory hierarchy is kept by the recruitment of one follicle from the group of 6–8 mm follicles on a near-daily basis [1].

The wall of the hen follicle in large part is composed of the extracellular matrix (ECM), which comprises collagenous fibers, dermatan sulfate, heparan sulfate, elastin, and hyaluronic acid. Furthermore, the basal lamina is rich in type IV collagen, fibronectin, and laminin. During ovulation, the basal lamina is completely degraded within the stigma region. In turn, during follicle atresia and POF regression, it disappears entirely. Hence, ECM turnover requires numerous proteolytic enzymes. Among them are matrix metalloproteinases (MMPs) [2,3,4,5], a disintegrin and metalloproteinases (ADAMs) [6], and ADAMs with thrombospondin motifs (ADAMTS) [7]. These proteins are capable of cleaving ECM macromolecules and non-ECM molecules [8,9,10,11].

The oocyte released upon ovulation of the F1 follicle is engulfed by the oviduct where components of the laid egg are synthesized and secreted, including the albumen, eggshell membranes, and eggshell. In addition to egg formation and transportation, the oviduct is the site of sperm storage and transport, fertilization, and early embryonic development. The mature hen oviduct consists of five morphologically and functionally distinguishable regions: the infundibulum (site of fertilization and production of the first layer of the egg white within 15–30 min), magnum (production of the majority of the egg white within 2–3 h), isthmus (creation of the inner and outer shell membranes within 1.5 h), shell gland (uterus; formation of the eggshell within about 19–20 h), and vagina (expulsion of the egg; sperm storage in sperm host glands) (Figure 1B).

## 3. MMP Family

The MMPs are a large family of zinc- and calcium-dependent enzymes that are divided into subgroups based on domain organization and the major ECM substrate preference, i.e., collagenases (MMP-1, MMP-8, MMP-13, MMP-18), gelatinases (MMP-2, MMP-9), stromelysins (MMP-3, MMP-10, MMP-11), matrilysins (MMP-7, MMP-26), membrane-associated MMPs (six MT-MMPs, e.g., MMP-14, MMP-16), and other MMPs, e.g., metalloelastase (MMP-12), epilysin (MMP-28), or enamelysin (MMP-20) [9,12,13]. The MMP family members have up to 40% primary structural similarity and they are labeled with numbers ranging from MMP-1 to MMP-28 [11,12].

These enzymes represent the key players in the molecular communication between cells and the ECM, and several of them can function inside the cellular compartments of diverse cell types [9,11]. Different mechanisms target various MMPs to intracellular localizations, including faulty secretion, alternative splicing, organelle localization signals, or reentry of secreted MMPs via diverse endocytosis mechanisms. So far, 11 MMPs are known to function intracellularly (e.g., MMP-2, MMP-7, MMP-9, MMP-14, and MMP-26), where they have both proteolytic and nonproteolytic functions [9,11]. Thus, MMPs regulate a diversity of signaling events in physiological and pathological processes. MMPs possess the ability to cleave a wide array of ECM components, including collagens, gelatins, elastin, laminin, and plasminogen, as well as activate other MMPs. These enzymes also process many non-ECM molecules such as signaling proteins, including Fas ligand, tumor necrosis factor α, amyloid protein precursor, syndecan 1, CD44 receptor, E-cadherin, chemokines and other cytokines, growth factors and their binding proteins. In turn, these factors modulate cell functions and behavior, including cell apoptosis, adhesion, migration, proliferation, differentiation, or immune cell recruitment during inflammatory processes [10,14,15,16,17]. The so far reported substrates of MMPs, in large part, were identified utilizing in vitro experiments and proteomic tools; therefore, it should be noted that not all of them have been confirmed to be physiological targets for specific MMP activity in vivo [10,18]. Furthermore, nonproteolytic functions of some MMPs include signal transduction and transcription factor activity (for more details see [9,10,11]).

The most widespread MMPs are gelatinases, which have substrate specificity for a broad spectrum of ECM constituents such as collagens (type IV, V, VII, X, XI), gelatins, elastin, fibronectin, laminin, and plasminogen and also for many non-ECM molecules including chemokines (e.g., CCL4, CCL7, CCL8, CCL13), cytokines (e.g., interleukin-1β, tumor necrosis factor α), growth factors and their binding proteins [10,15]. In addition, several substrates for intracellularly located MMP-2 and MMP-9 are identified, e.g., calponin-1, talin, troponin I, α-actin, connexin 43, poly-ADP-ribose polymerase 1, and heat shock protein 60 [11]. MMP-2 is the first MMP to be reported to play a key subcellular role, i.e., inside cardiac myocytes where it affects ischemia-reperfusion injury [19]. Collagenases are able to cleave different collagens into gelatin which becomes susceptible to tissue proteinases, as well as process other signaling molecules. For example, MMP-1 can modulate protease-activated receptor signaling in a cell-dependent manner [10]. Stromelysins and matrilysins possess the ability to degrade ECM constituents (e.g., collagens, aggrecan, fibronectin, laminin, elastin), other MMPs, growth factors, and cytokines. Thus, they may control cell–extracellular matrix interactions or growth factor bioavailability. For example, MMP-7, which is synthesized by epithelial cells and secreted apically, exhibits activity toward ligands, such as the Fas ligand, or receptors that transduce proapoptotic signals and may thus influence the susceptibility of cells to apoptosis [10,14,17]. Moreover, its overexpression is observed in many tumor types. MT-MMPs possess a single-pass transmembrane domain (MMP-14, MMP-15, MMP-16, and MMP-24) or a glycophosphatidylinositol (GPI) linkage (MMP-17 and MMP-25) that anchors these enzymes to the extracellular cell surface [18]. Among the numerous substrates of MT-MMPs, identified mainly in vitro, are ECM components and other MMPs, including proMMP-2 [16,18,20,21].

## 4. Inhibitors of MMPs

The activity of MMPs is regulated by a number of mechanisms at multiple levels: gene expression (by microRNAs, growth factors, inflammatory cytokines, or hormones), conversion from proMMPs (zymogens) to active enzymes (by convertases, serine proteinases, or other MMPs), or inhibition (by serum-borne α2-macroglobulin and tissue-derived inhibitors termed tissue inhibitors of metalloproteinases) [11,22,23,24,25]. There are four tissue inhibitors of metalloproteinases (TIMP-1, TIMP-2, TIMP-3, and TIMP-4), which act selectively on different MMPs. For instance, TIMP-1 has a high affinity for MMP-9 and collagenases, TIMP-2 preferentially binds to MMP-2, TIMP-3, similarly to TIMP-1, binds preferentially to MMP-9, whereas TIMP-4 acts similarly to TIMP-2. TIMPs form complexes with active MMPs leading to inhibition of their proteolytic activity [13,24]. Moreover, TIMP-2 in conjugation with MT-MMPs can regulate the specific site of activation of other MMPs, e.g., of MMP-2. TIMPs show tissue-specific constitutive or inducible expression. In addition to inhibition of MMPs, TIMPs take part in different biological processes including steroidogenesis, cell proliferation and differentiation [26,27,28]. For example, TIMP-3 is widely expressed in reproductive tissues where it plays a role in apoptosis and ovarian steroidogenesis [27]. The relationship of TIMPs with the MMP expression is the key to the balance of MMP activity, therefore, TIMPs are essential for maintaining ECM homeostasis. The intracellular activation of MMPs may also occur by diverse mechanisms such as pro-peptide cysteinyl sulfhydryl oxidation (S-glutathiolation or S-nitrosylation) by reactive oxygen–nitrogen species, phosphorylation/dephosphorylation, and proteolytic cleavage by furin, serine protease, itself, or other MMP. The final level of MMP regulation is the degradation of the protease, which is thought to be mediated through their specific endocytosis and degradation in the lysosome [11].

## 5. MMP Roles in Follicle Development and Atresia

**Figure 2:**
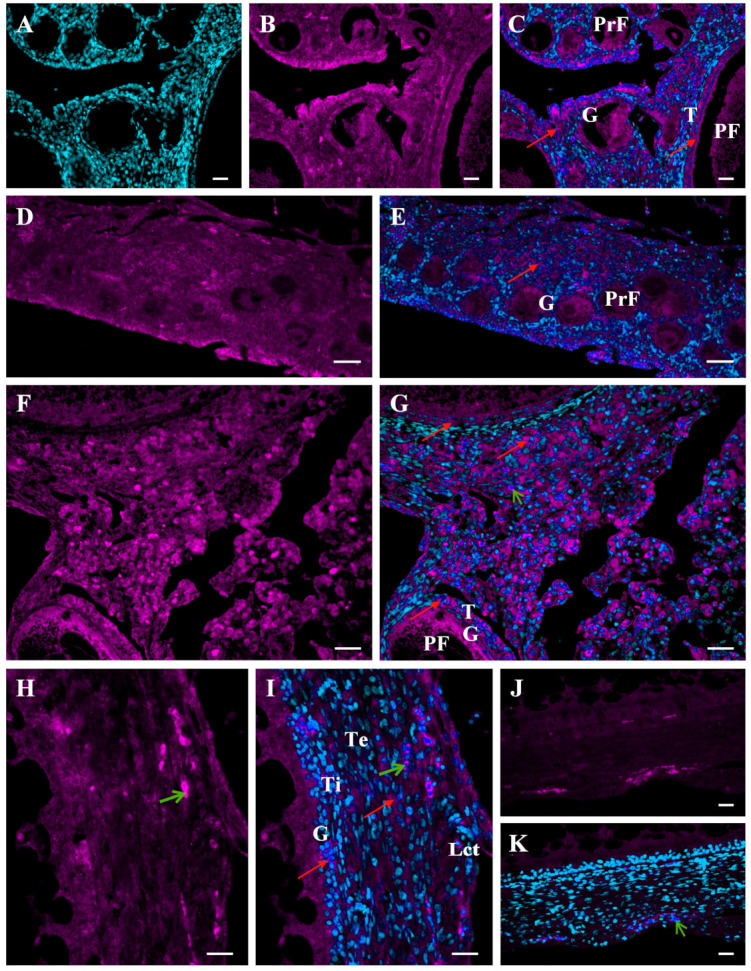
Immunofluorescent localization of the MMP-2 protein in the ovary of a growing chicken. Representative micrographs of the chicken ovary obtained about 6 weeks (**A**–**C**) and 4 weeks (**D**,**E**) before sexual maturation and of the ovarian stroma obtained from a mature hen (**F**,**G**). The wall of the yellowish follicle obtained from the ovary about two weeks before maturation (**H**–**K**). Positive fluorescence for MMP-2 (magenta; red arrows) present in ovarian stromal cells as well as the entire wall of primordial follicles (PrF; composed of one layer of granulosa cells), primary follicles (PF; composed of one layer of granulosa cells and a thin theca layer), and yellowish follicles (composed of several layers of granulosa cells, theca interna and externa, and epithelium with loose connective tissue). Negative control sections incubated without the primary antibody (Abcam, Cambridge, UK) do not exhibit positive staining (**J**,**K**), excluding red blood cells which always show nonspecific fluorescence (green arrows). DAPI staining (cyan) of the nucleus only (**A**). Specific immunoreactivity for MMP-2 (**B**,**D**,**F**,**H**). Merger of DAPI and MMP-2 (**C**,**E**,**G**,**I**,**K**). Abbreviations: G, granulosa; T, theca; Lct, epithelium with loose connective tissue; Ti, theca interna; Te, theca externa. Scale bars = 20 µm.

**Figure 3:**
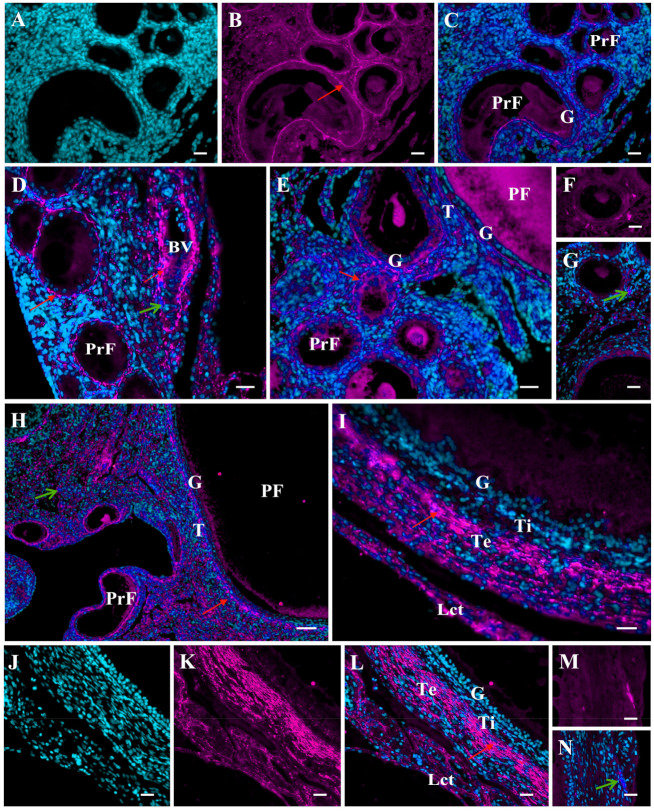
Immunofluorescent localization of the MMP-9 protein in the ovary of growing chickens. Representative micrographs of the chicken ovary obtained about six weeks (**A**–**C**,**F**,**G**), four weeks (**D**), and two weeks (**E**) before sexual maturation and of the ovarian stroma obtained from a mature hen (**H**). The wall of the white follicle (**I**) and the yellowish follicle (**J**–**N**) obtained from the ovary about four and two weeks before maturation, respectively. Positive fluorescence for MMP-9 (magenta; red arrows) presents extracellularly in the ovarian stroma, in the basal lamina surrounding primordial follicles (PrF), in the basal lamina and theca of primary follicles (PF), and primarily in the theca externa of white and yellowish follicles as well as around blood vessels (BV). Negative control sections incubated without the primary antibody (Abcam, Cambridge, UK) do not exhibit positive staining (**F**,**G**,**M**,**N**). DAPI staining (cyan) of the cell nuclei only (**A**,**J**). Specific immunoreactivity for MMP-9 (**B**,**K**). Merger of DAPI and MMP-9 (**C**–**E**,**G**–**I**,**L**,**N**). Other explanations and abbreviations as in Figure 2.

Ovarian follicle development in the avian ovary is a series of highly coordinated events such as recruitment of growing follicles into the preovulatory hierarchy or atresia and rupture of the follicular wall at ovulation. These processes require substantial remodeling of follicular wall architecture and changes in cellular physiology. The ECM is an essential contributor in establishing the developmental stage-specific follicle microenvironment that allows or restricts the access to growth factors and hormones for the follicle cells [13,22,29]. A growing body of evidence suggests that MMPs and inhibitors of MMPs play a relevant role in ECM remodeling of ovarian tissues [20,26,27,29,30,31,32,33]. Many MMPs are produced in the mammalian ovary and participate in the regulation of ovarian functions [20,29,31,33,34,35]; however, the role of MMPs in the avian ovary is not fully elucidated. So far, expression of several MMPs and their inhibitors has been shown in the chicken ovary. The mRNA and proteins of MMP-1, MMP-3, MMP-9, and MMP-13 have been detected in the juvenile and laying hen ovary [36,37]. The expression of these enzymes is higher in the ovaries of laying hens than in immature hens (23-week-old vs. 6-week-old and 16-week-old hens) indicating involvement of MMPs in prepubertal ovary development. Immunolocalization of MMP-2 and MMP-9 in ovarian tissues and within the follicular wall of ovarian follicles after their ontogenetic appearance in the developing ovary (Figure 2 and Figure 3) further suggests MMP involvement in ovary development during sexual maturation. MMP-2 immunoreactivity is present inside and outside the cells, and in the follicular wall, it is found in the granulosa and theca layers (Figure 2).

MMP-9 immunopositive reaction is observed clearly in the ECM, mainly around primordial and primary follicles, and in the wall of white and yellowish follicles it is detected primarily in the theca externa and the epithelium with loose connective tissue (Figure 3). The differential protein localizations and changes in mRNA and protein abundances of some MMPs may indicate their participation in the rapid ECM turnover occurring in the ovary during the last weeks before chicken sexual maturation. Namely, white follicles appear in the ovary about 3–4 weeks before maturation, and 2 weeks before the first ovulation, yellowish follicles are present in the ovary. In the last week before the onset of egg lay, tremendous growth of the yellow follicles occurs.

In follicles of the mature hen ovary, the expression of MMP-2, MMP-3, MMP-9, MMP-10, and MMP-13 mRNAs and/or proteins as well as gelatinase (MMP-2 and MMP-9) activities increase along with follicular maturation [36,38,39,40,41,42,43]. These correlative findings suggest the engagement of some MMPs in the turnover of the ECM/follicular wall structure during the final stage of ovarian follicle maturation. Moreover, transcript and/or protein abundances of MMP-2, MMP-7, MMP-9, MMP-10, and MMP-13 as well as of TIMP-2 and TIMP-3 differ between white and yellowish follicles and the small yellow follicles just recruited into the preovulatory hierarchy [39,40]. Furthermore, atresia of white follicles is accompanied by an increase in MMP-2 and MMP-9 activities [39]. These observations implicate the MMP system members in determination of the prehierarchical follicle destination, i.e., follicle atresia or selection to complete development.

An important phenomenon in the reproductive cycle of female birds is the regression of reproductive organs and the subsequent pause in laying and molting at the end of the reproductive season or after about a year of egg laying. This event leads to rejuvenation of reproductive tissues by the removal of old cells and proliferation and differentiation of new cells. During this period, yellow hierarchical follicles in the ovary undergo atresia. We show using quantitative real-time polymerase chain reaction (qRT-PCR) analysis of RNA from ovarian tissues that the expression of *MMP-2*, *MMP-9*, *MMP-10*, *MMP-13*, *TIMP-2*, and *TIMP-3* changes during the pause in egg laying induced by fasting, and these transcriptional alterations are reflected in protein abundance and activity of both gelatinases as measured by immunoblotting and the activity assay [42]. Advanced atresia of large yellow follicles characterized by pronounced disorganization of the wall structure is accompanied by decreased mRNA expression of examined MMPs and TIMP-3 concomitantly with increased *TIMP-2* mRNA levels. In turn, protein abundances of both gelatinases measured by Western blotting do not change or are decreased. For the activity of MMP-2 and MMP-9 as measured by the activity assay (Biotrak MMP Activity Assay System; GE Healthcare, Chalfont Saint Giles, UK), decreases are observed. These results indicate that MMPs may not be engaged in the final stage of atresia of yellow follicles in the avian ovary, while they may participate in the regulation of the early stage of follicle atresia. Such a role is suggested for gelatinases in the slightly atretic, prehierarchical follicles [39]. In addition, a decrease in MMP expression and activity noted simultaneously with advanced cell degradation may, at least in part, be related to caspase action. These enzymes are involved in degradation of a variety of proteins during apoptotic cell death in the hen follicular wall. Further investigation is warranted to define MMP expression and activity at different stages of follicle atresia in birds.

Importantly, all the MMPs detected are expressed more strongly in the theca than in the granulosa layer. In the latter, MMPs are at a very low level, barely detectable (e.g., MMP-9, MMP-13) or not detectable (e.g., MMP-7) [38,39,40,41,42,43]. These findings are consistent with the presence of fibroblasts, which are a known source of MMPs in the theca layer. Other sources of MMPs may be macrophages and mast cells, which are primarily frequent in the theca layer of the largest preovulatory follicles [44,45]. In addition, the theca layer in large part includes the ECM, which is composed of various target molecules for processing by different MMPs. Tissue- and cell-dependent localizations of several MMP proteins in chicken ovarian follicles [36,37,39,42] (Figure 2 and Figure 3) highlight distinct roles of different MMPs in these structures. MMP-2 protein is localized inside and outside cells through the entire follicular wall. MMP-9 is present exclusively in the ECM, mainly in the theca externa layer and loose connective tissue. Gelatinases have a similar proteolytic property, can bind denatured and native collagen; however, MMP-2 cleaves in vitro native collagen types I and II in a similar manner to collagenases, but MMP-9 does not [46]. One of the functions of these gelatinases may be degradation of ECM components and intercellular junctions. For example, MMP-2 as well as MMP-7 and MMP-9 may process the gap junction protein, connexin 43 [9]. Intracellular MMP-2 may mediate the mitochondrial membrane dysfunction [9] and lead to cell apoptosis, which is particularly intensified during follicle atresia. Abundant MMP-9 as well as MMP-13, predominantly around blood vessels, combined with proangiogenic roles of these MMPs [47], may be attributable to their functions in the development of the blood vessel network in the wall of growing follicles, which expands maximally in the largest F1 follicle. In turn, the function of MMP-7 may be related to cell–extracellular matrix interactions and molecule bioavailability.

The domestic hen ovary is an important model for human ovarian cancer. In both women and domestic hen, ovarian tumors have similar histological subtypes and cellular marker expression. Some data indicate that MMPs and their inhibitors are among them. Markedly increased abundance of the *MMP-3* transcript is found in chicken ovarian cancer and the expression pattern is relatively similar to that of MMPs in human cancer. Therefore, it suggests that the cell type-specific expression of the *MMP-3* gene renders it as a unique marker for epithelial ovarian cancer and indicates the significant role for MMP-3 in ovarian cancer development in hens [48]. Further, overexpression of α2-macroglobulin is observed in cancerous ovaries but not in normal ovaries of chickens; this may suggest this nonspecific MMP inhibitor is a critical regulator of epithelial cells of the hen ovary during its transition from the normal to a cancerous state [49].

## 6. MMP Roles in Ovulation

The process of ovulation includes a series of biochemical and morphological events occurring in parallel within the preovulatory F1 follicle which lead to its rupture along the stigma and release of the mature oocyte. The stigma area of the follicle becomes progressively thinner as a result of disintegration of the ECM by a cascade of proteolytic processes mediated by different proteases. A growing body of evidence has established that these events are largely controlled by MMPs. First, Nakajo et al. [50] suggested that proteolytic enzymes such as collagenases participate in follicular rupture in the chicken ovary. A marked increase in collagenase and acid and neutral protease activities is further observed immediately after ovulation in the POF1 follicle [51]. The presence of several MMPs in the basement membrane demarcating the theca and granulosa layers of chicken preovulatory follicles indicates their involvement in the breakdown of this membrane required to release the oocyte during ovulation [52]. Participation of gelatinases (MMP-2 and MMP-9) in follicular rupture is further supported by the higher expression and activity of both enzymes 3 h compared to 22 h before ovulation in chicken ovarian follicles as well as their markedly elevated activities in the F1 follicle compared to less mature follicles [39].

Ovulation is primarily orchestrated by pituitary-derived LH. This gonadotropin might initiate follicular production of enzymes competent to degrade the ECM components of the follicular wall including MMPs, and progressive decomposition of the wall results in its rupture. This assumption is supported by the findings of Zhu et al. [36] who observed FSH- and LH-stimulated in vitro *MMP-1* and *MMP-3* mRNA expression in the granulosa cells harvested from the preovulatory F4–F2 follicles. Simultaneously, *MMP-9* mRNA expression was not affected by LH or, at a high dose, was decreased. In turn, the study by Ogawa and Goto [53] revealed a stimulatory action of LH on acid proteinase and collagenase activities in tissue fragments of the stigma area of the preovulatory follicle. Furthermore, the results of an in vivo investigation demonstrate reduced activities of collagenase and neutral and acid proteinases in two of the largest preovulatory follicles from the ovaries of hypophysectomized chickens. These observations highlight that gonadotrophins, probably LH, are pivotal stimulators in the synthesis of enzymes that lead to follicle rupture during ovulation in hens [53].

It is interesting to note that ovulation resembles the inflammatory process [54], characterized by higher activity of some MMPs [10,16]. Thus, MMPs in addition to other inflammatory mediators may participate in the regulation of ovulatory events in the hen ovary. Another process (possibly related to ovulation), hatching in fish and amphibians, is also mediated by MMPs. Namely, Mmp13a has been shown as an important regulator of this event in zebrafish embryos [55].

## 7. MMPs in Postovulatory Follicles (POFs)

Unlike in mammals, the follicle in hens after ovulation fails to form a functional corpus luteum. Instead, the remaining follicular wall shrinks, becomes thickened, and the granulosa layer increases to several cells in thickness. The follicle terminates metabolic activity and undergoes rapid regression within approximately 4–6 days. During POF regression, the basal lamina disappears, the granulosa cells undergo lipid degeneration, and the theca interna and granulosa cells translocate towards the follicle lumen [56,57,58]. These events are accompanied by remarkable changes in the expression and activity of several MMPs. Leśniak and Hrabia [38] found increased abundance of the *MMP-2* transcript along with gradual regression of the chicken POF from POF1 to POF5. Next, Zhu et al. [36] showed increased mRNA and protein levels of MMP-1 and MMP-9 in the chicken POF1 compared to the F1 follicle. Recently, we demonstrated alterations in mRNA expression and immunoreactivity of MMP-2, MMP-7, and MMP-9 as well as activity of MMP-2 and MMP-9 in chicken POF1–POF5 during apoptotic regression of these follicles [58]. The highest activity of MMP-2 is noted in POF2 (about 26–45 h after F1 ovulation), whereas the activity of MMP-9 is the highest in POF2–POF4 (approximately 45–93 h after F1 ovulation). In addition, reduction in *TIMP-2* and *TIMP-3* expression is observed. These findings implicate MMP system proteins as important mediators in the complex orchestration of chicken POF regression, as is strongly suggested for the mammalian corpus luteum [34].

## 8. MMP Regulation in the Avian Reproductive System

### 8.1. MicroRNA-Mediated MMP Regulation

In the chicken ovarian follicles, several differentially expressed microRNAs (e.g., miR-31-5p, miR-31-3p, miR-92-3p, let-7d, and miR-138-1-3p) and their potential target genes (*MMP-1*, *MMP-3*, *MMP-10*, *MMP-11*, *MMP-16*, *MMP-25*, *ADAM10*, *ADAMDEC*, *ADAMTS8*, and *ADAMTS15*, *COL4A3*, and *COL4A6*) are evidenced because of their roles in maintaining and turnover of the ECM required for follicle development, ovulation, and atresia [59]. The target genes of the differentially expressed microRNAs are linked with four signaling pathways. As is revealed, miR-31-5p, miR-92-3p, let-7d, and miR-202-3p might regulate the pathway of inhibition of MMPs, ultimately leading to follicle growth, selection, or ovulation. The same microRNAs and their target genes of MMPs (*MMP-1*, *MMP-10*, and *MMP-16*) and ADAMs (*ADAM10*, *ADAMDEC*, *ADAMTS8*, and *ADAMTS15*) seem to be significant players in the axonal guidance signaling pathway, controlling processes occurring in the developing ovarian follicles of hens. By way of example, Mmp25β plays such a crucial role in the modulation of axon elongation in zebrafish during development [60]. Furthermore, the HIF1α signaling pathway with its implicated microRNAs (miR-31-5p, miR-92-3p, let-7d, miR-202-3p, and miR-449b) and their target MMP genes (*MMP-3*, *MMP-10*, *MMP-11*, and *MMP-25*) is indicated as significant in the regulation of follicle vasculature and ovulation [59]. In addition, the GP6 signaling pathway, with associated miR-92-3p, miR-31-5p, miR-202-3p, and let-7d and their target *ADAM10* and collagen IV genes, is proposed to be involved in ECM remodeling in the chicken ovary [59]. Collectively, these findings indicate that microRNAs are important regulators of MMP system protein expression.

### 8.2. Gonadotropin-Mediated MMP Regulation

Gonadotropins seem to be important regulators of MMP system proteins in the ovarian follicles of birds. Recently, the potential engagement of gonadotropins in the regulation of expression and activity of several MMPs as well as TIMP expression in ovarian follicles at different stages of development were evaluated in vivo in chickens [43]. For this purpose, laying hens were treated for several days with equine chorionic gonadotropin (eCG) which exerts a primarily FSH-like bioactivity in birds and has a stimulatory impact on follicle selection and growth of recruited follicles. We found that the effect of eCG on the expression and activity of selected members of the MMP system depends on the type of MMPs or TIMPs, the layer of the follicular wall, and the stage of follicle maturation. Overall, eCG acts as a negative regulator of MMP expression and activity in the largest hierarchical follicles. Based on these observations, it has been suggested that gonadotropins, principally FSH, regulate transcription, translation, and/or activity of MMP system constituents. In turn, these MMPs and TIMPs participate in the mechanisms that underlie ECM remodeling and cell function throughout ovarian follicle development in the chicken ovary [43]. As described in chapter 6, the LH may primarily stimulate the synthesis/activity of MMPs leading to follicle rupture during ovulation [53].

### 8.3. Steroid Hormone-Mediated MMP Regulation

Growing evidence indicates that steroid hormones are also important regulators of MMPs and their inhibitors in the chicken ovary. A study by Ogawa and Goto [53] demonstrated a stimulatory role of progesterone on neutral and acid proteinase and collagenase activities as well as promotion of the effect of estradiol on acid proteinase activity in cultured ovarian follicle walls of hens. Another in vitro investigation revealed that progesterone added to the culture medium of granulosa cells from the F4–F2 follicles promotes *MMP-1* and *MMP-3* mRNA expression but does not affect *MMP-9* mRNA [36]. Recently, in an in vivo study in which hens were treated with tamoxifen (estrogen receptor modulator), we showed a role for estrogen in regulating the expression and/or activity of several MMPs (MMP-2, MMP-9, MMP-10, MMP-13) and TIMPs (*TIMP-2* and *TIMP-3*) in ovarian follicles: white, yellowish, small yellow, and three of the largest yellow preovulatory ones [41].

### 8.4. Other Factors Involved in MMP Regulation

The author’s recent results [40] suggest that prolactin is another actor in the hen ovary implicated in the orchestration of ECM turnover and/or cell functions. Treatment of egg-laying hens with recombinant chicken prolactin induces alterations in the mRNA expression of *MMP-2*, *MMP-7*, *MMP-9*, *MMP-10*, *MMP-13*, *TIMP-2*, and *TIMP-3*, as well as in the protein abundance of gelatinases in different ovarian follicles. The relatively minor changes in gelatinase activity observed suggest that post-translational mechanisms play a major role in regulating MMP activity. Overall, prolactin seems to be a positive regulator of MMP expression in preovulatory follicles in the chicken ovary. Regulating the transcription, translation, and/or activity of some MMPs and TIMPs, prolactin may determine the growth, selection, atresia, or maturation of the ovarian follicle.

Based on the cell culture experiment, an additional factor, namely, transforming growth factor β1, has also been proposed to play a role in regulating the expression of *MMP-9* but not of *MMP-1* and *MMP-3* in granulosa cells of chicken preovulatory follicles [36].

## 9. MMPs in the Oviduct

In female embryos of chickens, initially, a pair of Müllerian ducts develop in the coelomic cavity and grow caudally until day 12 of incubation. Then, the right Müllerian duct undergoes gradual regression, and completely disappears by the time of hatching. At the same time, the left Müllerian duct develops into a functional oviduct [61] due to the promoting effect of estrogen and absence of the anti-Müllerian factor [62,63]. The changes occurring in the structural architecture of oviductal tissues during their development, regression, and rejuvenation require the controlled turnover of the stromal ECM. A growing body of evidence indicates that, as in other tissues, this process is regulated, at least in part, by proteolytic enzymes including MMPs and their inhibitors such as TIMPs and α2-macroglobulin. First, Ha et al. [64] provided evidence for MMP-2 involvement in the regression of the right Müllerian duct in the chicken embryo and demonstrated that estrogen (with its receptor α) prevents MMP-2 activity in the left Müllerian duct, leading to the failure of its regression. It should be noted that estrogen receptor α is primarily engaged in differentiation and development of the Müllerian duct [62,63]. In oviducts of growing and laying hens, the expression of several MMPs (MMP-2, MMP-7, MMP-9) and TIMPs (TIMP-2 and TIMP-3) as well as gelatinolytic activity occur [65,66,67,68]. The MMP and TIMP genes are differentially expressed in all oviductal sections on both mRNA and protein levels [65,66,67,68]. These differences in transcript and protein abundances of MMPs or TIMPs between the particular segments of the oviduct may be attributable to their specific tissue architecture, cell population, function, and physiological activity. During the maturation period characterized by tremendous changes in the morphology and function of the oviduct, transcript levels of several MMPs and TIMPs, abundances of the MMP-9 protein, and gelatinolytic activity decrease in most oviductal regions. Simultaneously, protein levels of MMP-2, MMP-7, and TIMP-3 increase, and TIMP-2 remains unaltered [66]. These findings indicate a role for MMPs and TIMPs in regulating the processes responsible for proper development of the chicken oviduct. Furthermore, regression and subsequent rejuvenation of the chicken oviduct is accompanied by an increase in the abundances of *MMP-2*, *MMP-7*, *MMP-9*, *TIMP-2*, and *TIMP-3* transcripts as well as an increase in MMP-2 and MMP-9 activity without alterations in the protein levels of these members of the MMP system [67]. A discrepancy between mRNA expression, protein abundance, and MMP activity may be caused by different transcriptional, post-transcriptional, translational, post-translational, and activation regulatory mechanisms. Nevertheless, changes in the expression/activity of selected members of the MMP system dependent on the stage of the reproductive cycle point to these MMPs and TIMPs being important regulators of oviductal tissue remodeling in birds [67]. Moreover, cell- and tissue-specific localization of MMPs and TIMPs in the wall of the chicken oviduct indicates distinct roles of particular MMPs in oviduct development, regression, and repair [66,67,68]. One of the functions of MMP-2 in the chicken oviduct, present mostly in the epithelium and the stroma (connective tissue + muscles), and of MMP-9 (localized outside the cells) may be the degradation of ECM constituents and intercellular junctions as well as the release of ECM molecules, leading to oviductal tissue regression, repair, development, or differentiation. MMP-2 localized inside muscle cells may cleave cytoskeletal proteins, leading to muscle degeneration during oviduct regression. For example, processing of junctophilin-2 by MMP-2 during myocardial ischemia-reperfusion injury results in cardiac muscle contractile dysfunction [69]. In turn, intracellular Mmp2 in the zebrafish striated muscle likely participates in the maintenance of sarcomere function [70]. Moreover, MMP-2 is likely secreted into the oviductal lumen becoming one of the components of the egg albumen. The latent MMP-2 in a complex with TIMP-2 is present in both the oviductal fluid and the egg white [65]. The localization of MMP-7 and TIMP-3 in the luminal epithelium and tubular glands in addition to their increased abundances just before sexual maturity may be attributable to the differentiation of tubular gland cells that appear in the oviductal wall at this time of development. Moreover, MMP-7 localization points to its possible role in cell apoptosis, which is especially intensified during oviduct regression [71,72]. Another study demonstrated α2-macroglobulin in the chicken oviduct, which is most abundant specifically in the luminal and glandular epithelium indicating involvement of this nonspecific MMP inhibitor in epithelial cell biology [49]. Although regulation of MMP system proteins remains largely unknown in the avian oviduct, the contribution of estrogen in the regulation of expression and/or activity of these molecules in the oviduct of immature [49] and mature [68] birds is proposed.

## 10. Conclusions

Collectively, the existing data advance our understanding of the mechanisms underlining regulation of MMP system protein expression/activity in the chicken reproductive system at different physiological states. Detailed mechanisms regulating synthesis and activity of MMP system members and their specific roles in particular processes occurring within the avian ovary and oviduct require further clarification.

## Data Availability

Not applicable.
